# L’hydronephrose géante idiopathique: vers une approche thérapeutique en 2 temps

**DOI:** 10.11604/pamj.2017.27.54.10385

**Published:** 2017-05-24

**Authors:** Taher Ismail Boudhaye, Mohamed Sidimalek, Cheikhani Jdoud

**Affiliations:** 1 Service d’Urologie, Hôpital Militaire de Nouakchott, Mauritanie; 2 Faculté de Médecine de Nouakchott, Mauritanie; 3 Service de Réanimation, Hôpital Militaire de Nouakchott, Mauritanie; 4 Service d’Urologie, Centre Hospitalier National, Mauritanie

**Keywords:** Hydronéphrose géante, syndrome de jonction, Giant hydronephrosis, ureteropelvic junction syndrome, CT scan, deferred nephrectomy

## Abstract

L’hydronéphrose géante est rare. Le syndrome de jonction pyélourétérale représente l’étiologie la plus fréquente. Nous rapportons un cas inhabituel d’un patient hospitalisé pour hydronéphrose géante avec altération de l’état général. Le diagnostic a été fait par le scanner. Le traitement avait consisté en une néphrectomie différée après drainage percutané.

## Abstract (en)

Giant hydronephrosis is rare. It is usually caused by ureteropelvic junction syndrome. We here report the unusual case of a patient hospitalized with giant hydronephrosis associated with impaired general condition. Diagnosis was based on CT scan. The patient underwent deferred nephrectomy after percutaneous drainage.

## Introduction

L’hydronéphrose géante est une entité rare. Elle est définie par une collection d’origine rénale occupant la moitié de l’abdomen ou le débordant sur la ligne médiane. Elle est souvent secondaire à une anomalie de la jonction pyélo-urétérale [1,2].

## Patient et observation

**Figure 1:**
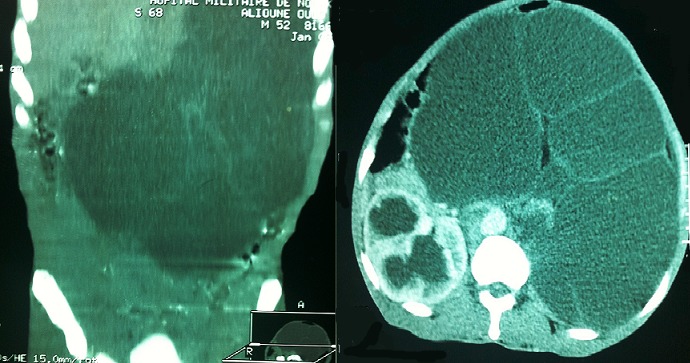
Uroscanner montrant une volumineuse hydronéphrose gauche, cloisonnée et compressive, occupant toute la cavité rétro péritonéale et s’étendant jusqu’au pelvis

**Figure 2:**
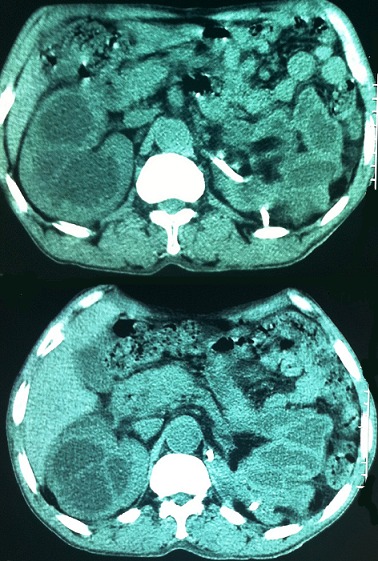
Uroscanner après mise en place de la néphrostomie montrant l’affaissement de l’hydronéphrose

**Figure 3:**
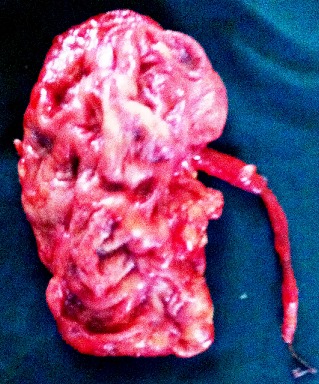
Pièce de néphrourétérectomie: le rein gauche est réduit à une poche multicloisonnée, l’histologie a conclu à des lésions de pyélonéphrite chronique non spécifique

Il s’agissait d’un patient âgé de 55ans, ayant été admis aux urgences pour des troubles digestifs évoluant dans un contexte d’altération de l’état général. L’examen a objectivé une énorme masse abdominale gauche, d’allure liquidienne, donnant le contact lombaire et débordant sur la ligne médiane. Le bilan morphologique, notamment l’Uroscanner a mis en évidence une volumineuse hydronéphrose gauche, compressive, occupant toute la cavité rétro péritonéale et s’étendant jusqu’au pelvis (Figure 1). Le patient a bénéficié initialement d’une néphrostomie percutanée, associée à une antibiothérapie pendant 1mois avec une bonne réponse clinique et radiologique (Figure 2). Le second temps de la prise en charge thérapeutique a consisté en une néphrectomie (Figure 3). L’évolution était marquée par une amélioration de l’état général et une reprise de l’activité au bout de quelques semaines.

## Discussion

L’hydronéphrose géante est une entité rare. Elle a été définie pour la première fois par Sterling en 1939 comme étant une collection d’urine dans les cavités excrétrices supérieure à 1 litre. En 1979, Crooks et al ont défini l’hydronéphrose géante comme une collection d’origine rénale occupant la moitié de l’abdomen ou le débordant sur la ligne médiane [2,3]. La cause la plus fréquente est l’obstruction de la jonction pyélourétérale, bien que la pathologie lithiasique, le traumatisme, l’ectopie rénale ainsi que les tumeurs urétérales ont été reportées [2].

Sur le plan clinique, elle se manifeste par une masse abdominale. Elle s’y associe des douleurs abdominales, des signes de compression digestive et parfois veineuse avec des œdèmes des membres inférieurs. L’uroscanner est l’examen morphologique clé, il permet le diagnostic positif et étiologique [1].

La néphrectomie serait la règle, associée à une réanimation et une antibiothérapie pour mise en condition du patient, en présence de stigmates de surinfection, l’abord chirurgical immédiat peux présager de nombreuses difficultés opératoires surtout sur des terrains multitarés, d’où l’indication du drainage percutané premier proposé par certains thérapeutes [1], cette attitude a été bien validée dans notre observation.

## Conclusion

Le drainage percutané avec néphrectomie différée devant une hydronéphrose géante représente une approche thérapeutique fiable, il permet une bonne récupération symptomatique et facilite l’abord chirurgical secondaire.

## Conflits d’intérêts

Les auteurs ne déclarent aucun conflit d'interêts.
